# Advancements in the Management of Postoperative Air Leak following Thoracic Surgery: From Traditional Practices to Innovative Therapies

**DOI:** 10.3390/medicina60050802

**Published:** 2024-05-13

**Authors:** Vasileios Leivaditis, Konstantinos Skevis, Francesk Mulita, Christos Tsalikidis, Athanasia Mitsala, Manfred Dahm, Konstantinos Grapatsas, Athanasios Papatriantafyllou, Konstantinos Markakis, Emmanuel Kefaloyannis, Glykeria Christou, Michail Pitiakoudis, Efstratios Koletsis

**Affiliations:** 1 Department of Cardiothoracic and Vascular Surgery, Westpfalz Klinikum, 67655 Kaiserslautern, Germany; vnleivaditis@gmail.com (V.L.); mdahm@westpfalz-klinikum.de (M.D.); thanospap9@yahoo.gr (A.P.); 2 Department of Thoracic Surgery, General Hospital of Rhodos, 85133 Rhodos, Greece; koskevis@gmail.com; 3 Department of General Surgery, Patras University Hospital, 26504 Patras, Greece; oknarfmulita@hotmail.com; 4 Second Department of Surgery, Democritus University of Thrace Medical School, 68100 Alexandroupolis, Greece; ctsaliki@med.duth.gr (C.T.); nancymits20@gmail.com (A.M.); 5 Department of Thoracic Surgery and Thoracic Endoscopy, Ruhrlandklinik, West German Lung Center, University Hospital Essen, University Duisburg-Essen, 45239 Essen, Germany; grapatsaskostas@gmail.com; 6 Department of Cardiothoracic Surgery, General Hospital of Nicosia, 2031 Nicosia, Cyprus; konsmarkakis@gmail.com; 7 Department of Thoracic Surgery, University Hospital of Heraklion, 71500 Heraklion, Greece; ekefaloyannis@uoc.gr; 8 Department of Thoracic Surgery, KAT Attica General Hospital, 14561 Athens, Greece; christouglykeria@gmail.com; 9 Department of Cardiothoracic Surgery, Patras University Hospital, 26504 Patras, Greece; ekoletsis@hotmail.com

**Keywords:** postoperative air leak, thoracic surgery, pulmonary complications, conservative management, surgical treatment, autologous plasma, pleural healing, chest tube management, pleurodesis

## Abstract

*Background:* Postoperative air leak (PAL) is a frequent and potentially serious complication following thoracic surgery, characterized by the persistent escape of air from the lung into the pleural space. It is associated with extended hospitalizations, increased morbidity, and elevated healthcare costs. Understanding the mechanisms, risk factors, and effective management strategies for PAL is crucial in improving surgical outcomes. Aim: This review seeks to synthesize all known data concerning PAL, including its etiology, risk factors, diagnostic approaches, and the range of available treatments from conservative measures to surgical interventions, with a special focus on the use of autologous plasma. *Materials and Methods:* A comprehensive literature search of databases such as PubMed, Cochrane Library, and Google Scholar was conducted for studies and reviews published on PAL following thoracic surgery. The selection criteria aimed to include articles that provided insights into the incidence, mechanisms, risk assessment, diagnostic methods, and treatment options for PAL. Special attention was given to studies detailing the use of autologous plasma in managing this complication. *Results:* PAL is influenced by a variety of patient-related, surgical, and perioperative factors. Diagnosis primarily relies on clinical observation and imaging, with severity assessments guiding management decisions. Conservative treatments, including chest tube management and physiotherapy, serve as the initial approach, while persistent leaks may necessitate surgical intervention. Autologous plasma has emerged as a promising treatment, offering a novel mechanism for enhancing pleural healing and reducing air leak duration, although evidence is still evolving. *Conclusions:* Effective management of PAL requires a multifaceted approach tailored to the individual patient’s needs and the specifics of their condition. Beyond the traditional treatment approaches, innovative treatment modalities offer the potential to improve outcomes for patients experiencing PAL after thoracic surgery. Further research is needed to optimize treatment protocols and integrate new therapies into clinical practice.

## 1. Introduction

Postoperative air leak (PAL) is a common and challenging complication encountered after thoracic surgery, encompassing a wide range of procedures from lung resection to pleural interventions [1,2]. Defined as the persistent escape of air from the lung into the pleural space, PAL poses significant clinical and economic implications, impacting patient recovery trajectories, prolonging hospital stays, and escalating healthcare costs. More than half of the patients who undergo lung resection experience an air leak within the first 24 h following the procedure [3,4]. PAL following lung resection is characterized by ongoing air escape from the remaining lung tissue after the 5th day post-surgery [2,3]. It is considered as the most frequent complication after pulmonary resection, with its occurrence estimated at between 5% and 25% [5]. The management of PAL remains a pivotal concern for thoracic surgeons and healthcare teams, demanding a nuanced understanding of its etiology, risk factors, and effective intervention strategies to mitigate its occurrence and expedite patient recovery [1,6].

The clinical significance of PAL extends beyond the immediate postoperative period, influencing the overall success of thoracic surgery procedures and patient quality of life. The presence of an air leak can lead to complications such as pneumothorax, infection, delayed pleural effusion and, in severe cases, respiratory failure. Consequently, the prompt detection and management of PAL are paramount in the postoperative care of thoracic surgery patients [1,2,6].

The objectives of this review are twofold. Firstly, to provide a comprehensive overview of the current understanding of PAL, including its pathophysiology, contributing risk factors, and the methodologies employed in its diagnosis and monitoring. Such a foundation is essential for the effective management and treatment of PAL. Secondly, this review aims to delineate the spectrum of management strategies available, ranging from conservative measures to surgical interventions. Some innovative treatments have garnered attention for their potential to reduce the incidence and duration of PAL, representing novel approaches in the arsenal against this postoperative complication.

In pursuing these objectives, this review synthesizes existing literature, clinical guidelines, and recent studies to present a narrative that is both informative and pragmatic. By elucidating the complexities surrounding PAL and the diverse strategies employed in its management, this article endeavors to equip healthcare professionals with the knowledge necessary to optimize patient outcomes following thoracic surgery.

## 2. Materials and Methods

To construct a comprehensive review of PAL following thoracic surgery, a systematic literature search was performed across multiple electronic databases, including PubMed, Cochrane Library, and Google Scholar. The search was conducted to include publications from 2000 to the present, giving more emphasis to the articles published in the past decade and capturing the most recent and relevant advances in the field.

**Selection Criteria**: The inclusion criteria for articles were meticulously defined to ensure the inclusion of high-quality and pertinent data. The criteria were as follows:

- *Type of Publications:* Peer-reviewed research articles, reviews, clinical guidelines, and meta-analyses.
- *Language:* Articles published in English.
- *Relevance:* Publications that specifically addressed the incidence, pathophysiology, diagnostic approaches, and management strategies for PAL.
- *Innovative Treatments:* Studies that detailed emerging therapies and techniques.
- *Study Outcomes:* Articles that provided clear outcomes of treatment efficacy, patient recovery timelines, and any complications associated with different management strategies.

The search strategy included the use of specific keywords and phrases aligned with our review’s aims, such as “postoperative air leak” “thoracic surgery” “pleural healing” “autologous plasma” and “innovative treatments in PAL management”.

**Article Selection Process**: Initially, titles and abstracts were screened for relevance based on the inclusion criteria. Relevant articles underwent a full-text review to further assess their suitability for inclusion in our review. This two-step filtering process ensured a rigorous selection of sources, enabling a thorough and balanced discussion of PAL.

By methodically outlining our selection criteria and the scope of the publication years, we aimed to provide a robust foundation for our review, ensuring that our conclusions are based on comprehensive and current evidence.

## 3. Etiology and Risk Factors

Understanding the etiology and risk factors associated with PAL is crucial for its prevention and management. PAL occurs when there is an abnormal passage for air between the alveoli and pleural space, creating a bronchopleural fistula (BPF) which persists beyond the immediate postoperative period. This section delves into the pathophysiological mechanisms and identifies the key risk factors that contribute to the development of PAL [1].

### 3.1. Pathophysiology

PAL occurs when there is an abnormal and persistent communication between the alveoli and the pleural space, resulting in a bronchopleural fistula (BPF). This condition primarily arises from the disruption of lung parenchyma integrity, which can occur due to surgical incisions, stapling, or tissue handling during thoracic procedures. Despite the lung’s inherent ability to seal minor air leaks through tissue adhesion and pleural healing, these mechanisms can be overwhelmed, especially in the presence of underlying pulmonary conditions or extensive surgical manipulation [2,7].

Factors contributing to the risk of PAL include the incomplete lung expansion post-surgery, as well as the presence of bullae and blebs often encountered in surgeries for conditions like pneumothorax and emphysema. As described by Cerfolio et al., the severity of postoperative air leaks is influenced by the phase of the respiratory cycle in which they occur, making their management a critical aspect of postoperative care (Table 1) [3].

### 3.2. Risk Factors

Understanding the underlying risk factors is crucial for clinicians seeking to identify patients at higher risk of PAL and to implement targeted preventive measures [8]. Preoperative optimization of lung function, careful surgical planning, and technique selection based on individual patient risk profiles are essential components of minimizing the incidence of PAL [4,6,7,8,9,10,11,12]. The risk factors can be divided into patient-related and surgical or perioperative factors. Those two risk factor groups are separately presented in Table 2 and Table 3. These tables highlight the multifactorial nature of PAL risk, demonstrating the importance of a comprehensive approach to patient assessment and management to mitigate this postoperative complication.

Risk scoring systems, such as those developed by Brunelli, Epithor, and the European Society of Thoracic Surgeons (ESTS), play a crucial role in predicting the likelihood of PAL after thoracic surgery [10,13,14]. These systems integrate various patient-specific factors to assess risk, facilitating preoperative planning and personalized patient care. The Brunelli score considers gender, age, body mass index (BMI), forced expiratory volume in 1 s (FEV_1_) less than 80%, and the presence of pleural adhesion [10]. The Epithor score expands on these criteria, incorporating gender, the surgical site’s location, dyspnea score, BMI, type of resection, and pleural adhesion [13]. Meanwhile, the ESTS score simplifies the model to include gender, BMI, and FEV_1_. By quantifying the risk of prolonged air leak, these scoring systems enable surgeons to identify high-risk patients, potentially guiding the selection of surgical techniques and postoperative management strategies to mitigate this complication [14].

## 4. Diagnosis and Monitoring of Postoperative Air Leak

The diagnosis and effective monitoring of postoperative air leak (PAL) are critical components in the management of patients undergoing thoracic surgery. Timely identification and assessment of PAL severity allow for appropriate interventions to be implemented, thus minimizing potential complications and facilitating recovery. This section explores the methodologies employed in the diagnosis and monitoring of PAL, highlighting the integration of clinical assessment with technological advancements [1,2,15].

### 4.1. Clinical Assessment

The initial diagnosis of PAL primarily relies on clinical observation, including signs such as unexpected or prolonged chest tube bubbling. Clinicians monitor symptoms including dyspnea, chest pain, and reduced oxygen saturation, which suggest compromised lung function. Physical signs such as subcutaneous emphysema, detectable by a crackling sensation upon palpation, or changes in voice tone, are critical for early detection. While these clinical and patient-reported indicators are crucial, they must be confirmed with more objective diagnostic methods [2,15,16].

### 4.2. Imaging Techniques

Chest X-ray (CRX) is crucial for postoperative assessment, visualizing lung re-expansion and significant air collections indicative of persistent air leaks, though it is less sensitive for detecting small leaks [2,15]. For more detailed imaging, computed tomography (CT) or magnetic resonance imaging (MRI) scans are used to identify smaller and more precisely located air leaks. However, their use is limited by higher costs and availability, making them suitable primarily for complex cases or when CRX results are inconclusive [1,2].

### 4.3. Intraoperative Detection

Intraoperative detection of air leaks aims to reduce postoperative complications by identifying leaks during surgery. The standard method, the water submersion test (WST), involves filling the thoracic cavity with saline to observe air bubbles during manual positive pressure ventilation, noted for its subjectivity and variability based on the surgeon’s experience (Table 4). This technique is especially challenging in video-assisted thoracoscopic (VATS) procedures due to limited visibility and the manipulation required. Air leaks frequently occur near the fissures, away from suture lines, complicating efforts to seal them due to proximity to pulmonary artery branches [17,18]. A newer approach by Yang and Chang involves using surfactant in VATS for better visualization and detection of air leaks, proving to be safe and cost-effective [19].

Another technique for identifying air leak sites using low-pressure carbon dioxide (CO_2_) insufflation during thoracoscopic surgery has been shown to be safe, effective, and practical [20]. More advanced techniques, including the use of intraoperative fluorescence imaging after the administration of indocyanine green, are also being explored regarding their potential to enhance leak detection [21,22].

### 4.4. Monitoring Protocols

Effective PAL management requires standardized monitoring protocols to guide the assessment and intervention strategies. These protocols typically involve regular evaluations of air leak presence and volume, chest tube output, and patient respiratory status. Adjustments to patient care, including the decision to remove chest tubes or escalate treatment, are based on these systematic assessments, combined with clinical judgment [2,15,16].

The diagnosis and monitoring of PAL involve a blend of clinical assessment, imaging, intraoperative detection, and quantitative measurement. The integration of these methodologies enables a comprehensive approach to managing PAL, ensuring that interventions are timely, appropriate, and tailored to the individual patient’s needs. Continuous advancements in diagnostic and monitoring technologies hold the promise of further enhancing the accuracy and efficiency of PAL management in thoracic surgery [1,15].

## 5. Intraoperative Prevention of Air Leaks

Intraoperative prevention of air leaks is a critical focus in thoracic surgery, aimed at minimizing postoperative complications and facilitating patient recovery. Several methods have been developed and implemented to achieve this goal, including the use of reinforced staplers, the application of biological sealants, and the placement of autologous fat pads [2]. Reinforced staplers, often combined with materials such as bovine pericardium and polyglycolic acid, provide enhanced sealing of lung tissue at resection margins, significantly reducing the likelihood of air leakage [5,23]. Biological sealants, which may include fibrin glues or synthetic polymers, act to further seal the surgical sites and support tissue healing [24,25]. Additionally, the use of autologous fat pads, harvested from the patient, can be applied to cover and protect vulnerable areas, aiding in the prevention of PAL through natural tissue integration and healing [26].

The fissureless technique in lung surgery involves avoiding dissection along the pulmonary fissures, instead approaching the lung parenchyma and vessels from outside the fissures [27]. This method reduces the risk of air leaks by minimizing direct manipulation and potential damage to the fragile lung tissue and parenchymal air spaces often encountered in emphysematous or diseased lungs. By preserving the integrity of the lung parenchyma and reducing the exposure of raw surfaces, the fissureless technique decreases the likelihood of creating new pathways for air to escape, thereby lowering the incidence of postoperative air leaks [27,28].

Exploration of less conventional techniques has also contributed to advances in PAL prevention with promising outcomes. The use of the Thelium laser system to complete fissures offers precise tissue dissection and sealing, minimizing raw surface areas prone to air leaks [29]. Cryoneuroablation of the phrenic nerve aims to reduce diaphragmatic movement and the resultant residual space post-lung resection, thereby lowering the risk of PAL [30]. Decaluwe’s tunnel technique for fissure-first lobectomy in patients with incomplete fissures provides a strategic approach to resecting lung tissue while preserving integrity and reducing potential sites for air leaks [31]. These innovative techniques represent the ongoing evolution in thoracic surgical practices, emphasizing the importance of intraoperative strategies in preventing PAL and enhancing patient outcomes.

## 6. Conservative Management of Postoperative Air Leaks

Conservative management strategies are foundational in the treatment of PAL, particularly for minor leaks that are expected to resolve spontaneously. These approaches focus on optimizing chest drainage, promoting lung expansion, and enhancing patient comfort while the lung heals [2,5]. This section outlines the key conservative methods used in managing PAL, emphasizing their roles, effectiveness, and considerations.

### 6.1. Chest Tube Management

Chest tube management remains the cornerstone of conservative PAL treatment. The primary goal is to ensure adequate drainage of air and fluid from the pleural space, thereby facilitating lung re-expansion and sealing of the leak [2,15]. The approach to managing chest drains for postoperative air leaks varies significantly, with different methodologies focusing on the application of suction, the timing, and the level of negative pressure used, alongside the potential benefits of utilizing digital drainage systems [32]. Brunelli et al. have indicated that an individualized, regulated suction strategy, particularly when applied overnight within a range of −11 to −20 cm H_2_O, may facilitate patient mobility during the day and potentially shorten air leak durations when compared to traditional water seal methods, though these findings have not consistently reached statistical significance [4,33]. Contrarily, Alphonso et al. have supported a no-suction policy, advocating for a more conservative approach [34]. Further investigations, including a randomized controlled trial published by Holbek et al., revealed that low suction (−10 cm H_2_O) notably decreased the duration of chest drainage in comparison to water seal [35]. Additionally, retrospective analyses reported by Mitsui et al. have suggested that a low-pressure suction (−10 cm H_2_O) is more effective at improving postoperative air leaks than lower negative pressures (−20 cm H_2_O) [36]. These divergent findings highlight the ongoing debate and need for conclusive research to establish standardized guidelines for chest drain management following thoracic surgery to minimize air leak durations [2,15]. Moreover, the decision to clamp the chest tube prior to removal is made with caution, often involving a trial period to assess if the air leak has adequately resolved without the risk of tension pneumothorax [15,37].

Although the conventional method often still involves the use of two chest drains after major lung resection, managing an air leak with just one drain has been found to be sufficient [15,38]. There is no evidence to suggest that using two drains offers any advantages over a single drain. The current body of research supports the adequacy of a single chest drain, indicating that it may reduce both the time needed for chest drainage and the length of hospital stay [38,39,40]. Should a patient’s air leak not be effectively managed by one drain, a second may be required, yet there is no evidence to show that starting with two drains decreases the likelihood of needing to insert an additional drain later [15,38,41].

### 6.2. Application of External Suction

The debate over the use of external suction versus not using it for managing air leaks persists, with two main physiological theories put forward [15,42]. The first theory argues that external suction could hinder the healing of air leaks by encouraging airflow through the fistula, suggesting that air leaks might heal quicker without the use of suction. On the other hand, an opposing viewpoint posits that suction helps by drawing the parietal and visceral layers of the pleura together, thereby aiding in the closure of the air leak [15,43]. Despite several meta-analyses presenting varied perspectives, the choice to use suction is often made at the institutional level. It is generally believed that adding suction to a simple water-seal system does not significantly affect the duration of air leaks, hospital stay lengths, or the incidence of prolonged air leaks after lung surgery [42,43,44,45,46].

### 6.3. Optimizing Pulmonary Function

Enhancing pulmonary function through physiotherapy and incentive spirometry is critical in PAL management. These interventions aim to improve lung volume and encourage effective cough mechanics, which facilitate the closure of air leaks. Pulmonary physiotherapy may include techniques such as deep breathing exercises, cough encouragement, and the use of positive expiratory pressure devices. These measures not only aid in leak resolution but also prevent atelectasis and improve overall respiratory function [2,5,32,47].

### 6.4. Pain Management

Effective pain control is integral to the conservative management of PAL. Pain can inhibit deep breathing and effective coughing, thereby hindering lung expansion and prolonging the duration of air leaks. A multimodal pain management approach, including the use of analgesics, regional anesthesia techniques such as intercostal nerve blocks or epidural anesthesia, and non-pharmacological methods, can significantly improve patient comfort and respiratory effort [48,49].

### 6.5. Observation and Digital Air Leak Monitoring

Patients with minor PAL require close observation, including regular assessments of respiratory status, chest tube output, and air leak measurement. This vigilant monitoring ensures timely identification of any deterioration in the patient’s condition, allowing for prompt escalation to more invasive interventions if necessary. Modern digital chest drainage (DCD) systems have revolutionized this aspect of care by providing precise, real-time measurements of air leak rates and pleural pressure, enabling a more tailored management approach [50,51]. These systems facilitate informed clinical decisions regarding chest tube management, such as the timing of chest tube removal and adjustments to suction pressure, which can optimize drainage and potentially accelerate leak resolution [52,53].

The effectiveness of digital versus traditional chest drainage systems has been extensively evaluated, with varying outcomes highlighted across several studies. Arai et al. did not find significant differences in postoperative outcomes between DCD and standard drainage systems, underlining the non-inferiority of digital systems and their benefits, such as portability and quiet operation [54]. Conversely, Comacchio et al. reported that DCD systems significantly shortened both chest drainage duration and hospital stays in VATS lobectomy patients, suggesting specific advantages in certain surgical contexts [55]. Similarly, Yagi et al. found that digital systems were particularly effective, significantly reducing the duration of chest drainage compared to analog systems [56]. In a broader review, Aldaghlawi et al. noted that DCD systems generally allow for shorter durations of chest tube usage and hospital stays, although the results varied and indicated the need for further research to confirm these findings across different patient groups [57].

Additional studies reinforce these findings but also present some inconsistencies. Wang et al.’s meta-analysis highlighted that DCD systems reduced the risk of prolonged air leaks and shortened recovery times in pulmonary resection patients, potentially enhancing patient outcomes and quality of life [58]. Yet, Takamochi et al. did not observe significant differences in chest tube placement duration or hospitalization between digital and analog systems in their randomized study of patients undergoing anatomic lung resection [59]. Moreover, Gilbert et al. noted that while DCD systems reduced the need for chest tube clamping trials, they did not significantly affect the overall duration of chest tube drainage or hospital stay after stratifying by postoperative air leak status, indicating that the benefits of digital technology might be limited to specific patient subsets [53]. Marulli et al. aimed to further clarify these aspects by comparing electronic and traditional systems in a multicenter trial, focusing on interobserver variability and the differentiation of active air leaks from pleural space effects [60]. Finally, Zhou et al. and Lee et al. suggested that the DCD system primarily offers benefits in reducing the duration of chest tube placement, shortening hospital stays, determining the optimal timing of chest tube removal and therefore decreasing the frequency of chest tube clamping tests, but future studies should investigate the practical and financial implications of routinely implementing digital systems in clinical settings [61,62]. Overall, these studies suggest that while digital chest drainage systems offer several advantages, including more precise monitoring and potentially reduced hospital stays, their effectiveness and utility can vary based on the surgical and patient-specific contexts. The transition to digital systems seems promising but requires careful consideration of clinical needs, costs, and potential benefits as evidenced by ongoing research and varied clinical outcomes across studies.

Ambulatory chest drainage systems equipped with one-way valves, such as the Heimlich valve, a digital system, or a vacuum bottle represent a significant advancement in the management of PAL, offering patients greater mobility and comfort during recovery [63]. These compact, portable devices allow air to escape from the pleural space without re-entry, effectively managing air leaks while preventing the accumulation of air or fluid that could lead to tension pneumothorax. The use of a one-way valve system enables patients to be discharged earlier from the hospital and continue their recovery at home, reducing healthcare costs and improving patient satisfaction. This approach not only facilitates a more active postoperative period but also supports the psychological well-being of patients by allowing them to return to their daily routines more quickly. These devices are particularly beneficial for managing PAL when traditional chest tube management would otherwise require prolonged hospitalization [63,64,65,66]. Moreover, Dinjens supported that treatment of PAL in an ambulatory setting using a digital monitoring device achieved a high success rate with minimal complications [67].

Conservative management of PAL emphasizes a patient-centered approach, integrating various supportive strategies to facilitate natural healing processes [32]. While effective for many patients, especially those with minor leaks, it requires careful monitoring and a readiness to adapt the management plan based on the patient’s evolving clinical status. Through judicious application of these conservative methods, many patients with PAL can achieve successful resolution without the need for surgical intervention.

## 7. Pleurodesis

### 7.1. Chemical Pleurodesis

Chemical pleurodesis is a targeted intervention for the management of PAL, wherein a sclerosing agent, such as talc, doxycycline, povidone iodine or bleomycin, is administered into the pleural cavity to induce pleural adhesion [68]. This procedure aims to obliterate the space between the lung and chest wall, thereby preventing further air leaks by promoting the fusion of the visceral and parietal pleura [69]. After the sclerosing agent is applied intrapleurally, several reactions occur within the pleural space: widespread inflammation, an imbalance between coagulation and fibrinolysis that leans towards fibrin adhesion formation, the attraction and growth of fibroblasts, and increased collagen production. The sclerosant primarily affects the pleural mesothelial lining, which is crucial in the entire pleurodesis process. This includes initiating the release of various mediators such as interleukin-8, transforming growth factor-β, and basic fibroblast growth factor, all playing key roles in the procedure [70]. Applied through thoracoscopy or via a chest tube, chemical pleurodesis offers a practical solution for PAL that persists despite conventional treatments, helping to stabilize the patient’s condition and facilitate recovery. Its effectiveness in sealing the pleural space and preventing the recurrence of air leaks makes it a valuable option in the comprehensive management of PAL [32,69].

Pleurodesis using glucose solutions represents a less commonly employed but effective method for managing recurrent pleural effusions and postoperative air leaks. This technique involves instilling hypertonic glucose solutions into the pleural cavity, which works by inducing an osmotic gradient that promotes fluid reabsorption and leads to pleural irritation [71]. The resulting inflammatory response encourages the fusion of the visceral and parietal pleura. While this method is advantageous for its simplicity, low cost and the low risk of adverse reactions associated with glucose, its application is carefully considered against other sclerosing agents, depending on the patient’s specific condition and overall treatment goals [71,72].

The choice of sclerosant and method can significantly impact its efficacy and safety profile. Talc pleurodesis, as shown in studies by Manger et al. [73] and Shaw and Agarwal [74], is highly effective with an 85% success rate, although concerns about discomfort during the procedure and potential for serious complications such as respiratory distress remain. Alternatively, Damaraju et al. [75] and Park et al. [76] compared iodopovidone and doxycycline, respectively, finding similar efficacy to talc but with potentially fewer severe adverse effects, suggesting a safer profile, particularly in patients at higher risk of complications. The use of glucose solutions, as explored by Talebzadeh et al. [77], presents a less common but viable alternative, offering comparable success rates to more traditional agents like bleomycin but with potentially less discomfort and cost. Moreover, the study by Jabłoński et al. [78] demonstrates the effectiveness of iodine in pleurodesis, showing favorable outcomes in terms of reduced hospital stay and pleurodesis duration compared to other agents, with minimal side effects. This contrasts with findings from Ong et al. [79], where talc was superior in preventing recurrence but associated with higher rates of fever post-procedure. Bresticker et al. emphasized that while mechanical methods like abrasion remain effective, the simplicity and efficacy of talc make it a preferable option in many cases [80]. These varied findings highlight the importance of tailoring pleurodesis techniques to individual patient needs and clinical scenarios, balancing efficacy, safety, and patient comfort to optimize outcomes.

### 7.2. Autologous Blood Patch

This technique stands as a globally recognized method for sealing PAL due to its numerous benefits [81,82,83]. It is notably advantageous because it can be administered bedside, repeated approximately every 48 h, carries no risk of allergic reactions, and has a low risk of adverse events or complications. Given the current evidence, it is not feasible to provide formal guidelines on executing the procedure. Randomized controlled trials are essential to verify its advantages [84]. The procedure, however, usually involves the injection of 50–120 mL of the patient’s own venous blood through the chest tube. Following administration, the chest tube is raised above the thorax insertion point to prevent early blood reflux. To ensure even distribution of blood in the pleural space, the patient is advised to rotate sides every 15 min for around two hours. This method leverages the natural clotting and sealing properties of autologous blood, offering a simple yet effective approach to managing PAL [2,81,85].

## 8. Surgical Treatment of Postoperative Air Leaks

For cases of PAL that persist despite conservative management, or are severe from the outset, surgical intervention may be necessary to achieve resolution. Surgical treatment aims to directly address the source of the air leak, restore lung integrity, and prevent recurrence [1]. Re-operation is infrequently needed and tends to be most beneficial when a significant air leak is unexpectedly discovered within the first 24 h following a lung resection [15,86].

In this section, the variety of surgical techniques available for managing postoperative air leaks when conservative measures are inadequate are explored. It is important to note that there is currently a lack of comprehensive studies comparing these techniques directly. Consequently, the selection of a surgical method should be tailored to the individual patient, taking into account the specific etiology of the air leak and the patient’s overall clinical context. This approach ensures that each patient receives the most appropriate and effective treatment.

### 8.1. Mechanical Pleurodesis

Mechanical pleurodesis, involving manual abrasion or partial to subtotal and total pleurectomy, is a surgical technique aimed at preventing recurrent postoperative air leaks and pleural effusions by physically creating adhesions between the lung and chest wall [87]. Manual abrasion pleurodesis is performed by directly rubbing the parietal pleura with a surgical instrument or gauze, inducing an inflammatory response that leads to pleural fusion [88]. In more extensive cases, a partial or total pleurectomy may be conducted, which involves the surgical removal of sections or the entirety of the parietal pleura, thereby eliminating the pleural space and fostering adhesion of the lung to the chest wall. These mechanical methods are particularly useful for patients with recurrent or persistent air leaks, offering a durable solution by effectively sealing the pleural cavity and minimizing the risk of recurrent future leaks [70,89].

### 8.2. Surgical Repair

Direct surgical repair targets the specific site of the air leak. Techniques vary based on the leak’s location and cause, ranging from simple suture repair of lung parenchyma to more complex procedures like resection of non-viable lung tissue. VATS is often preferred due to its minimally invasive nature, allowing for precise identification and repair of the leak site with reduced postoperative pain and faster recovery compared to open thoracotomy [86,90]. Bronchoscopy is recommended to determine whether the air leak originates from a bronchial fistula rather than lung parenchyma. If the remaining lung tissue is largely unaffected, re-stapling or suturing the leak often yields positive outcomes. Additionally, decortication might be necessary to enable the lung to fully expand [86].

### 8.3. Reinforcement Techniques

To enhance the durability of the repair and reduce the risk of recurrence, surgeons may employ reinforcement techniques. These can include the application of biological glues or sealants that promote tissue adhesion and healing at the repair site. Autologous materials, such as blood patches or fibrin glue, and synthetic sealants are used to create a seal over the leak, providing additional support to the lung tissue as it heals [6,91]. The pleural tent is a surgical technique used to reduce postoperative air leaks by creating an adhesion between the parietal and visceral pleura, thereby promoting natural sealing of the pleural space. This elevation of the pleura reduces the dead space in the pleural cavity and helps the lung to expand against the chest wall, facilitating the adherence of the lung to the chest wall and promoting the sealing of air leaks [92].

### 8.4. Bullectomy

In cases where PAL is associated with the presence of bullae or blebs, a bullectomy may be performed. This procedure involves the excision of these air-filled sacs from the lung surface, thereby eliminating the source of the leak. Bullectomy can be conducted via VATS or open thoracotomy, depending on the extent and location of the bullae [6,86,90].

### 8.5. Lobectomy

For extensive or complex air leaks that cannot be resolved through localized repair or in the presence of significant underlying lung disease, a lobectomy may be considered. This radical approach is reserved for situations where the benefits of removing diseased or damaged lung tissue outweigh the risks of surgery [6,86,90].

### 8.6. Thoracoplasty

Thoracoplasty, or the creation of an open window thoracostomy, is a seldom-used surgical intervention reserved for extreme cases of prolonged postoperative air leakage that do not respond to conventional treatments. This invasive procedure involves the surgical removal of rib segments to collapse the space where the air leak persists, or creating a direct external opening to the pleural space to facilitate drainage and healing. Such measures are considered only when all other options have been exhausted, reflecting the procedure’s significant nature and the complexities associated with managing persistent air leaks. The rarity of its use demonstrates the importance of exploring all less invasive options before proceeding to such drastic measures [86].

### 8.7. Postoperative Care and Monitoring

Following surgical intervention for PAL, meticulous postoperative care is essential to ensure successful outcomes. This includes ongoing chest tube management, pain control, respiratory support, and surveillance for complications. Regular imaging and clinical assessments guide the postoperative management plan, including the timing of chest tube removal and discharge planning [1].

## 9. Innovative Treatments of Postoperative Air Leaks

As the field of thoracic surgery continues to evolve, innovative treatments have emerged for managing PAL, focusing on enhancing the body’s natural healing processes and reducing the need for invasive interventions. Among these, the use of autologous plasma has garnered significant attention for its potential in treating PAL.

### 9.1. Autologous Plasma

Employing plasma for pneumothorax treatment represents an alternative and highly effective pleurodesis method [93]. The use of fresh frozen plasma (FFP) as an alternative to the traditional blood patch has been documented. Administering FFP intrapleurally, followed by patient rotation movements, ensures its even distribution. This process allows plasma to cover the surgically affected area of the visceral pleura, triggering a coagulation cascade akin to intravenous injection. This leads to the formation of a fibrin scaffold over the area of the lung that is leaking air within just a few hours. Combined with the local bioactive factors from the alveolar epithelium, this method not only seals the air leak but also offers protection against infections [94,95].

Autologous plasma therapy involves the application of a patient’s own plasma, rich in growth factors and platelets. This technique leverages the plasma’s natural healing properties to promote tissue repair and seal the leak more effectively. The process typically involves collecting a small amount of the patient’s blood, centrifuging it to separate the plasma, and then applying the plasma directly to the lung tissue or using it in conjunction with a biological glue [93,96]. Early research and clinical experiences suggest that autologous plasma can accelerate the resolution of PAL, reduce hospital stays, and decrease the need for further surgical intervention. Although it has not been assessed through large-scale, randomized studies, plasma instillation has demonstrated reliability as a pleurodesis technique, with minimal to nonexistent complications. Evidence regarding the use of plasma indicates both a lack of associated adverse effects and high efficacy in resolving air leaks. However, more extensive studies are required to fully understand its efficacy and optimal application protocols [94,95].

### 9.2. Platelet Gel

The use of autologous platelet gel (APG) in managing PLA represents a novel and promising approach. This technique involves applying a gel, derived from the patient’s own platelets, directly to the site of the lung that has been surgically treated [97]. The APG promotes healing by releasing growth factors that stimulate tissue repair and regeneration. Its autologous nature significantly reduces the risk of allergic reactions and complications related to foreign substances. Initial findings suggest that APG can effectively seal air leaks, potentially reducing the duration of hospital stays and improving patient outcomes by harnessing the body’s natural healing mechanisms. However, the experience with APG in this context remains limited, and there is a clear need for larger-scale studies to fully understand its efficacy and potential benefits in the treatment of postoperative air leaks [97,98].

### 9.3. Bioengineered Tissue Sealants

Advancements in bioengineering have led to the development of novel tissue sealants and adhesives designed to mimic the body’s natural healing mechanisms [99]. These products, often based on synthetic or biologically derived materials, aim to provide immediate sealing of air leaks while promoting tissue regeneration [6]. Some of these sealants are engineered to be biodegradable, gradually being absorbed by the body as the lung tissue heals.

The “lung-mimetic” sealant, a hydrofoam material, mimics the lung’s structure and properties with its alveolar-like porous ultrastructure, lung-like viscoelasticity (adhesive, compressive, tensile), and contains lung extracellular matrix-derived signals (matrikines) to aid in tissue repair. Biocompatibility tests indicate the sealant has minimal cytotoxicity and immunogenicity. When exposed to the sealant’s matrikines, human primary monocytes in vitro activate genes associated with pleural wound healing and tissue repair. In animal models, the sealant effectively closes air leaks and returns lung mechanics to normal levels. Their application ranges from minimally invasive procedures to open thoracotomy, offering a versatile tool in the surgeon’s arsenal against PAL [99].

Moreover, new sealant materials, including alginate methacrylate and gelatin methacryloyl, have been developed, each enhanced through the addition of dopamine HCl. These compounds can be cross-linked to form hydrogel patches for pre-application or create hydrogels directly at the injury site, using FDA-approved photo-initiators and oxidants. These sealants are easy to apply, non-toxic, and have shown promising results in both in vitro and in vivo models of lung and tracheal injuries. Nonetheless, clinical studies are essential to thoroughly assess their safety and effectiveness for medical use [100].

### 9.4. Endobronchial Valves

Originally developed for the treatment of severe emphysema, endobronchial valves have found a new application in managing PAL. These one-way valves can be placed bronchoscopically to redirect airflow away from the leak site, allowing the affected lung area to rest and heal. The procedure for bronchial valve implantation is thoroughly documented and consists of three main phases: (i) pinpointing the specific segment or subsegment of the bronchial tree responsible for the air leak, achieved by sequential balloon inflations while an indwelling chest drain is in place and monitoring for the air leak to cease; (ii) selecting the correct valve size with the help of manufacturer-provided sizers; and (iii) the actual placement of the valve [101,102]. This non-surgical approach has shown promise in selected cases, particularly for patients with localized air leaks who are poor candidates for further surgery. The temporary use of endobronchial valves provides a minimally invasive option to control PAL, with the potential for removal once healing is confirmed [103]. However, information on using this treatment for air leaks is primarily confined to a limited number of case series, with only a few studies specifically addressing postoperative air leaks [104].

### 9.5. Stem Cell Therapy

Over the past two decades, cell therapy and tissue engineering have become increasingly significant in treating diseases that lack effective cures, with numerous studies investigating cell therapy’s potential across various conditions [105]. Specifically, in treating pulmonary disorders, mesenchymal stem cells (MSC) present a new and hopeful approach due to their abilities in immunomodulation, tissue regeneration, enhancing bacterial clearance, and their proangiogenic and antifibrotic characteristics. Emerging research into stem cell therapy offers a novel approach to treating PAL. The theory behind stem cell therapy is to harness the regenerative capabilities of MSCs to repair damaged lung tissue and seal air leaks. While still in the experimental stages, preliminary studies suggest that MSCs can be directed to the site of lung injury, where they facilitate healing and tissue regeneration [105,106]. Preclinical studies have documented the impact of human adipose-derived stem cells on the regeneration of injured mesothelial cells of the visceral pleura in animal models [107,108]. Limited clinical data imply that employing autologous MSCs in high-risk patients seems practical, safe, and shows promising efficacy [109]. This approach represents a cutting-edge frontier in PAL management, with ongoing studies needed to validate its effectiveness and safety.

All these approaches offer hope for more effective, less invasive management options, with the potential to significantly improve patient outcomes. As research progresses and these technologies are refined, they may become integral components of the thoracic surgeon’s toolkit for managing PAL.

## 10. Discussion

The management of PAL after thoracic surgery presents a dynamic challenge, demanding a nuanced approach that blends technological advances with traditional clinical wisdom [1]. This review synthesizes recent research and clinical experiences to understand better the multifaceted nature of PAL management. The main articles included in this review are presented in Table 5.

The complexity of PAL begins with its etiology and risk factors, which are deeply integrated into the patient’s clinical profile and the surgical environment. Studies such as those by Sridhar et al. [6], Zheng et al. [7] and Zaraka et al. [9] have demonstrated that factors including patient age, underlying pulmonary pathology, and the extent of surgical resection significantly influence PAL risk. This variability underpins the necessity for personalized risk assessment and tailored intervention strategies to effectively mitigate PAL occurrence [2,5].

Advances in diagnostic technologies, particularly DCD systems, have revolutionized the monitoring and management of PAL. These systems provide real-time data on air leak dynamics, which crucially inform the management strategy. However, as pointed out, the reliance on such technology can also highlight healthcare disparities, underscoring the need for accessible solutions in resource-limited settings [50,51,52].

The decision between conservative and surgical management of PAL hinges on a careful evaluation of the leak’s persistence and severity. While conservative methods remain the first line of defense, their effectiveness can be limited by the nature of the air leak. Evidence from the current literature has shown that early surgical intervention may be warranted in cases of high-output leaks where conservative management fails to reduce the air leak within the first postoperative days [2,5,32,48,49,64,69].

When surgery is required, techniques such as VATS offer a minimally invasive option with a lower morbidity profile, which is particularly valuable in vulnerable patient populations. Comparative studies have demonstrated that VATS can reduce hospital stay and improve postoperative pain, compared to traditional open thoracotomy [6,15,86,87,90]. The choice of surgical technique is guided by a thorough preoperative evaluation and intraoperative findings, aiming to achieve the best possible outcomes while minimizing the risk of recurrence [1,15,86]. Collaboration between thoracic surgeons, anesthesiologists, and pulmonologists is crucial to optimize patient care throughout the surgical and recovery process.

The exploration of novel therapies such as autologous plasma, bioengineered tissue sealants, and stem cell therapy is expanding the boundaries of PAL management. These treatments aim to harness the body’s natural healing mechanisms, offering potentially less invasive alternatives to traditional methods. However, the clinical adoption of these innovations requires robust validation through controlled trials to ensure they provide a real benefit in clinical practice [99,101,107,108].

Moving forward, the field must address the disparities in access to care and explore how new technologies can be integrated into routine practice without exacerbating existing healthcare inequalities. Ongoing research should aim to refine treatment algorithms and continue the development of interventions that can be customized to patient-specific factors.

Overall, the management of PAL is characterized by a complex interplay of factors requiring comprehensive clinical judgment, advanced technological support, and a deep understanding of each patient’s unique medical background. The future of PAL management lies in an evidence-based, patient-centered approach that leverages both innovative treatments and proven strategies to improve outcomes for patients undergoing thoracic surgery.

## 11. Future Directions

This review aims to underline the significance of personalized patient care, the crucial role of technology in diagnosis and management, and the critical importance of ongoing research to refine and validate new treatment methodologies. As the field advances, it remains imperative for clinicians to stay abreast of emerging therapies, integrating evidence-based innovations into practice to enhance patient outcomes. The journey towards optimizing the management of postoperative air leaks is ongoing, fueled by a dedication to surgical excellence and patient-centered care. By embracing a comprehensive, evidence-based approach and remaining committed to the exploration of new frontiers in treatment, the thoracic surgical community can continue to improve the quality of life for patients.

## 12. Conclusions

The management of PAL following thoracic surgery encompasses a broad spectrum of strategies, from the fundamental to the innovative, each tailored to address the nuances of this complex complication. Our comprehensive review has highlighted the importance of a multifaceted approach that begins with a thorough understanding of PAL’s etiology and risk factors, progresses through meticulous diagnosis and monitoring, and culminates in the application of both conservative and surgical treatment modalities. The advent of innovative treatments, particularly the use of autologous plasma, alongside bioengineered tissue sealants, endobronchial valves, and the potential of stem cell therapy, heralds a new era in the management of PAL, promising more effective and less invasive options for patients.

## Figures and Tables

**Table 1:** Classification of postoperative air leak severity according to Cerfolio et al. [3].

Classification	Description
Grade 1	Air leak observed only at forced expiration or cough
Grade 2	Air leak observed with normal expiration
Grade 3	Air leak observed with normal inspiration
Grade 4	Air leak observed throughout the respiratory cycle but ceases momentarily
Grade 5	Continuous air leak observed throughout the entire respiratory cycle without cessation

**Table 2:** Patient-related risk factors for PAL [1,4,6,7,8,9,10,11,12]. This table outlines the patient-specific factors that have been identified to increase the risk of developing PAL following thoracic surgery. It provides insights into how each factor contributes to the risk, highlighting the importance of individualized patient assessment and management to mitigate PAL occurrence.

Patient-Related Risk Factors	
Risk Factor	Description
Chronic Lung Diseases	Conditions such as COPD, emphysema, or pulmonary fibrosis increase risk.
Smoking History	Smoking adversely affects the integrity of lung parenchyma.
Tumor Stage	Advanced stages often require more extensive surgical resections, potentially increasing the vulnerability of lung tissue.
Age and Nutritional Status	Elderly patients and those with poor nutritional status may have reduced tissue repair capabilities.
Obesity	Obesity can lead to increased surgical complications.
Genetic Predisposition	Genetic factors may influence lung tissue integrity and the ability to heal.
Preoperative Pulmonary Function	Lower preoperative lung function can indicate a higher risk of PAL.
Immunosuppression	Patients with weakened immune systems may have delayed healing.
Diabetes Mellitus	Diabetes can impair wound healing and lung recovery.

**Table 3:** Surgical and perioperative risk factors for PAL [1,4,6,7,8,9,10,11,12]. This table details the surgical and perioperative factors that contribute to the risk of PAL. It emphasizes the influence of surgical technique, procedure type, and intraoperative considerations on PAL incidence. Understanding these factors is essential for surgical teams seeking to plan and execute thoracic surgeries with minimized risk of PAL.

Surgical and Perioperative Risk Factors	
Risk Factor	Description
Type of Surgery	Certain procedures, like lung resections and lung volume reduction surgery for emphysema treatment, have a higher incidence of PAL.
Upper Lobe Resections	Upper lobe resections carry a higher risk of air leak due to their greater elastic recoil, sparser anastomotic blood supply, prevalence of emphysematous changes in the upper lobes, and the technical challenges associated with their proximity to major airways and vessels.
Pleural Adhesions	They complicate surgical dissection, potentially leading to inadvertent lung parenchyma injury and disruption of the lung surface integrity during surgery.
Surgical Technique	Use of certain instruments and techniques can affect tissue integrity.
Intraoperative Lung Manipulation	Excessive handling or manipulation of lung tissue can predispose to PAL.
Extended Operative Time	Longer surgeries may increase the risk of PAL due to prolonged exposure.
Anesthesia Type	Specific types of anesthesia may affect lung function.
Postoperative Pain Management	Inadequate pain management can prevent effective coughing and deep breathing.
Mechanical Ventilation Use	Use of mechanical ventilation can affect lung mechanics.
Experience of Surgical Team	Less experienced surgical teams may cause higher rates of PAL due to technique variability.

**Table 4:** Air leak score classification based on the intraoperative water submersion test [17,18].

Grade	Description
0	No leak
1	Countable bubbles
2	Stream of bubbles
3	Coalesced bubbles

**Table 5:** The main articles cited in this study.

Ref.	Authors	Year	Study Focus	Key Findings	Relevance to PAL Management
[1]	Geraci TC, et al.	2021	Postoperative air leaks in lung surgery	Identified predictors, intraoperative techniques, and management strategies.	Insights into comprehensive management of PAL.
[2]	Aprile V, et al.	2023	Conservative management of PAL	Reviewed intraoperative prevention and conservative management strategies.	Highlights the importance of non-surgical management.
[3]	Cerfolio RJ, et al.	1998	Management algorithm for air leaks post-pulmonary resection	Developed a prospective management algorithm.	Basis for many current protocols; emphasizes early management.
[4]	Brunelli A, et al.	2004	Predictors of prolonged air leak after lobectomy	Investigated patient and surgical factors influencing air leak duration.	Identifies risk factors to inform surgical planning.
[6]	Sridhar P, et al.	2020	Prevention of postoperative prolonged air leaks	Explored preventive measures for PAL after pulmonary resection.	Offers insights into effective preventive strategies.
[10]	Brunelli A, et al.	2010	Scoring system to predict risk of prolonged air leaks	Introduced a scoring system based on patient-specific factors.	Helps in preoperative planning and risk assessment.
[13]	Orsini B, et al.	2015	Validation of prolonged air leaks score in VATS	Validated a scoring system for predicting PAL risk in VATS procedures.	Assists in assessing risk and planning VATS procedures.
[15]	French DG, et al.	2018	Management of parenchymal air leaks	Discussed optimal management techniques for parenchymal air leaks.	Provides guidelines for effective postoperative care.
[19]	Yang HC, et al.	2018	Novel air leak test using surfactant for lung surgery	Introduced a novel test for detecting air leaks during surgery.	Enhances intraoperative detection and management.
[20]	Kang DY	2020	Intraoperative air leak detection via CO_2_ insufflation	Demonstrated safe and practical air leak site detection during surgery.	Improves intraoperative detection and potential outcomes.
[21]	Okusanya OT, et al.	2018	Infrared intraoperative fluorescence imaging	Explored the use of indocyanine green for intraoperative leak detection.	Offers advanced technique for enhancing leak detection.
[24]	Lequaglie C, et al.	2012	Use of sealant to prevent prolonged air leaks	Evaluated the effectiveness of sealants in preventing prolonged air leaks.	Supports the use of biological sealants in surgery.
[27]	Li SJ, et al.	2017	Fissureless technique for decreasing PAL	Reviewed the efficacy of avoiding pulmonary fissure dissection.	Suggests techniques that minimize surgical air leaks.
[33]	Brunelli A, et al.	2013	Tailored suction in chest drains	Compared tailored suction strategies for managing air leaks.	Influences chest drain management to reduce PAL duration.
[34]	Alphonso N, et al.	2005	Suction vs. non-suction to underwater seal drains	Evaluated the impact of suction on underwater seal drains post-lung resection.	Questions the necessity of suction in drain management.
[35]	Holbek BL, et al.	2019	Effects of low suction on digital drainage devices	Investigated the impact of low suction settings on digital drainage devices.	Supports refined suction strategies in postoperative care.
[48]	Marshall K, et al.	2020	Pain management in thoracic surgery	Reviewed methods for effective pain management in thoracic surgeries.	Emphasizes the importance of pain management in PAL recovery.
[50]	Filosso PL, et al.	2010	Digital air leak monitoring	Reviewed the impact of digital monitoring systems on managing air leaks.	Highlights advancements in monitoring technologies.
[54]	Arai H, et al.	2018	Evaluation of digital drainage systems	Compared outcomes with digital vs. traditional drainage systems.	Demonstrates benefits of digital systems in clinical practice.
[56]	Yagi S, et al.	2022	Clinical utility of digital vs. analog drainage systems	Digital systems reduced the duration of chest drainage compared to analog.	Shows efficacy of digital systems in managing PAL.
[62]	Lee SA, et al.	2021	Digital thoracic drainage systems	Evaluated clinical application of digital systems for air leak management.	Highlights precision and quantification advantages of digital systems.
[64]	Joshi JM	2009	Ambulatory chest drainage	Discussed the efficacy and safety of ambulatory systems for managing PAL.	Advocates for patient mobility and comfort during recovery.
[68]	Liberman M, et al.	2010	Persistent air leak management with chemical pleurodesis	Reviewed incidence and risk factors for persistent air leaks and pleurodesis use.	Highlights chemical pleurodesis as a valuable management option.
[70]	Park EH, et al.	2019	Doxycycline vs. talc for chemical pleurodesis	Compared the efficacy and safety of doxycycline and talc in pleurodesis.	Offers insights into safer pleurodesis options.
[77]	Talebzadeh H, et al.	2023	Glucose solution for pleurodesis	Compared 50% glucose solution to bleomycin for pleurodesis efficacy.	Suggests less common but effective pleurodesis agents.
[81]	Hugen N, et al.	2022	Autologous blood patch for prolonged air leaks	Systematic review on the efficacy of autologous blood patch for PAL.	Validates the effectiveness and safety of the blood patch method.
[93]	Skevis K, et al.	2022	Plasma pleurodesis	Explored fresh frozen plasma as an alternative pleurodesis method.	Introduces novel applications of plasma in pleurodesis.
[97]	Andreetti C, et al.	2010	Autologous platelet gel for persistent air leaks	Investigated the efficacy of platelet gel in sealing air leaks post-lung resection.	Suggests a novel and less invasive option for managing PAL.
[99]	Pinezich MR, et al.	2024	Lung-mimetic hydrofoam sealant	Developed a biodegradable sealant that mimics lung tissue for treating air leaks.	Represents innovative bioengineering approach to PAL management.
[101]	Mahajan AK, et al.	2013	Use of endobronchial valves for persistent air leaks	Demonstrated the effectiveness of endobronchial valves in managing persistent air leaks post-thoracic surgery.	Introduces a minimally invasive method to control PAL, enhancing recovery and reducing the need for further surgical intervention.
[105]	Chen X, et al.	2021	Stem cell therapy for pulmonary disorders	Reviewed potential of mesenchymal stem cells in treating pulmonary disorders including PAL.	Emerging research into regenerative options for PAL treatment.

## Data Availability

The authors confirm that the data supporting this study’s results are available within the present review.
